# Stability and validity of intact parathyroid hormone levels in different sample types and storage conditions

**DOI:** 10.1002/jcla.23771

**Published:** 2021-04-01

**Authors:** Haitham Khalil, Anwar Borai, Mohammed Dakhakhni, Suhad Bahijri, Hala Faizo, Fawzi F. Bokhari, Gordon Ferns, Ahmed A. Mirza

**Affiliations:** ^1^ King Abdullah International Medical Research Center King Saud bin Abdulaziz University for Health Sciences, Pathology King Abdulaziz Medical City Jeddah Saudi Arabia; ^2^ Department of Medical Laboratory Technology Faculty of Applied Medical Sciences King Abdulaziz University Jeddah Saudi Arabia; ^3^ Department of Clinical Biochemistry Faculty of Medicine King Abdulaziz University Jeddah Saudi Arabia; ^4^ Academic Affairs, Armed Forces Hospital Administration Taif Saudi Arabia; ^5^ Division of Medical Education Brighton and Sussex Medical School Mayfield House Brighton United Kingdom

**Keywords:** intact parathyroid hormone, pre‐analytical, stability, storage, temperature, validation

## Abstract

**Background:**

Several pre‐analytical factors can affect the measurement of intact Parathyroid Hormone (IPTH). In this study, we have investigated the effects of using different types of tubes, time elapsed before separation, and storage conditions over time on the measured values of IPTH.

**Method:**

Blood samples from 30 subjects were collected into plain, SST, and EDTA tubes. All serum and plasma were separated immediately (first set) and after 2 hrs delay (second set). The first set of samples were aliquoted and stored at RT (25°C), at fridge (4°C), and freezer (−20°C). IPTH was measured in all the stored aliquots at 2,4, and 8 days after collection using Architect analyzer.

**Results:**

Paired T test and ANOVA repeated measures showed no significant difference between IPTH levels in all tubes. The second set of serum and plasma were significantly lower (3.8% and 7.4%, *p *< 0.001, respectively) when compared to samples measured initially. Serum samples stored at RT were significantly lower (by 45%,59%, and 77%) on days 2,4, and 8 when compared to the initial time (*p *< 0.001 in all cases). Plasma samples stored at RT, were significantly lower on day 8 after collection, by 30.8% (*p *< 0.001). These differences would be clinically important.

**Conclusion:**

Plasma IPTH can be stored at RT for up to four days. Both plasma and serum IPTH are not affected by a delay in the separation of up to two h and they can be stored for up to 8 days in a fridge or freezer without any clinically significant changes in their values.

## Graphical abstract

Intact Parathyroid Hormone (IPTH) can be affected by different pre‐analytical factors. In this study, we investigated the effect of different types of tubes, delay in sample separation, and different storage temperature on the measured IPTH values. We found that plasma IPTH can be stored at RT for up to four days. Both plasma and serum IPTH are not affected by a delay in separation of up to two h and they can be stored for up to 8 days in a fridge or freezer without any clinically significant changes in their values.

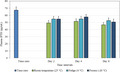

## 1 INTRODUCTION

The parathyroid glands are responsible for maintaining blood calcium level; this occurs by their secretion of parathyroid hormone (PTH) which exerts its effect on bone, kidney, and gastrointestinal system to maintain calcium level.^1^

Whilst many blood analytes are quite stable, and their measurements remain unaltered in different conditions, others are sensitive to several factors including time of sample collection, tube type, sample transportation, and sample storage conditions. Parathyroid hormone is critically important in clinical diagnosis of serum calcium abnormalities, and it has been shown that measured PTH levels vary widely between different commercial kits due to the lack of PTH standardization.^2^ The optimum procedure to measure IPTH is rapid centrifugation after blood collection, followed by immediate measurement. This cannot always be guaranteed in many laboratories, however, due to factors such as test availability, equipment readiness, batching analysis, and distance from clinics to the laboratory, etc. Such delays could affect the stability of the IPTH, leading to measurement errors that may, in turn, affect the treatment of patients.

It has shown that about 70% of the laboratory errors are pre‐analytical.^3^ The issue of pre‐analytical stability has been widely investigated in different biological analytes in order to establish the appropriate procedures to give reliable results. Therefore, a number of studies have sought to identify the factors affecting the pre‐analytical stability of IPTH in order to help develop more appropriate procedures but these have had inconsistent outcomes, are outdated, and/or contradict each other in respect to optimum storage conditions (see the summary in Table 1). Moreover, previous studies have tended to focus on individual factors, such as sample type (serum vs. plasma),^4^, ^5^ storage temperature,^6^, ^7^ and storage time.^8^ No study has attempted to evaluate all the major factors in the same protocol.

**TABLE 1:** Summary of previous studies on IPTH stability

Publication	Aim	Analyzer	Recommendations
Omer H. et al., 2001^4^	To investigate the difference between measured PTH in serum and EDTA plasma in different conditions.	IMMULITE 2000 (Siemens Healthcare Diagnostics, Munich, Germany)	PTH is more stable in EDTA plasma than serum. The presence of a few discrepancies, however, showed that it is not completely stable.
Glendinning P. et al., 2002^5^	To investigate the difference between intact PTH results carried out from serum separated tube (SST) and EDTA plasma.	IMMULITE 2000 (Siemens Healthcare Diagnostics, Munich, Germany)	EDTA plasma IPTH is higher than that in serum at collection time. After 3 days, serum result decreased by 60% compared to that at collection. In contrast, IPTH in EDTA plasma after 3 days had not changed from the measurement at the time of collection.
Teal T. et al.,2003 ^8^	To assess the stability of IPTH in EDTA plasma.	IMMULITE 2000 (Siemens Healthcare Diagnostics, Munich, Germany).	A time delay before freezing was associated with a significant decrease in IPTH concentration in serum in plain tubes (after two h) and in serum in SSTs (after 4 h). Plasma in EDTA tubes was not affected up to 48 h.
Cavalier E. et al.,2007^6^	To investigate the difference between IPTH in EDTA and serum in different conditions.	Roche Elecsys assay.	There was no significant difference in degradation between EDTA and serum after 1 day in frozen samples. Serum IPTH is more stable at −20°C for 5 days EDTA IPTH is more stable at room temperature.
Lau'ulu S. et. al.,2010 ^7^	To evaluate the differences in IPTH results in different collection tubes.	Beckman Coulter, (Fullerton, CA, USA). ARCHITECT i2000SR (Abbott Diagnostics Abbott Park, IL, USA) ADVIA Centaur (Siemens Healthcare Diagnostics, Deerfield, IL, USA) Modular E170 (Roche Diagnostics, Indianapolis, IN, USA) IMMULITE 2000 (Siemens Healthcare Diagnostics) LIAISON (DiaSorin, Stillwater, ‐‐‐‐‐MN, USA)	Frozen and refrigerated samples were statistically different when compared with fresh samples. No clinically significant difference between serum and plasma IPTH in fresh samples or 24 h refrigerated samples.
Hanon E. et al., 2013 ^16^	To review previous studies to devise optimum conditions of pre‐analytical procedure in measuring IPTH.	Systematic review of previous studies, no listed instruments	IPTH is more stable in plasma EDTA than serum at room temperature when RBCs or clots are immediately separated after collection. Decreasing IPTH values were observed in samples where there had been a two‐hour delay in the separation of serum from clots, even if separation was optimal. IPTH is stable in EDTA plasma up to 72 h and in serum up to 24 h at 4°C.
Schleck M. et al., 2017^17^	To evaluate the effect of the tube type on IPTH when blood samples are stored uncentrifuged after collection for 4 h and 18 h at 4°C and 25°C.	Roche Cobas (Roche, Basel, Switzerland) LIAISON XL (DiaSorin, Saluggia, Italy)	IPTH is more stable in plasma EDTA than serum at RT when samples were kept uncentrifuged for 18 h. An SST is recommended if not stored as whole blood for 18 h.

The aim of this study, therefore, is to investigate the following major factors contributing to variations in measured IPTH levels: the use of different types of blood collection tubes, the time elapsed before separation, and storage conditions over time.

## 2 MATERIALS AND METHODS

### 2.1 Study design

There were 30 participants in total: 20 male and 10 female healthy‐adult subjects. The mean ± SD of their age was 33.7 ± 9.0 years and BMI 26.2 ± 2.2 kg/m^2^, and of their systolic and diastolic blood pressure, 124 ± 14 mmHg and 73 ± 10 mmHg, respectively. In order to maintain consistent pre‐analytical conditions between different samples, blood samples were collected into five tubes (5 mL each) from each participant on the morning of the same day. Specifically, one Serum Separated Tube (SST II Advanced; gel tube without additives; Ref # 367955), two plain (without gel or additives; Ref # 368815), and two ethylene diamine tetra‐acetic acid (EDTA‐K2; Ref #8516333) tubes. All blood samples were collected using Becton‐Dickinson (BD) vacutainer tubes (Becton, Dickinson and Company Franklin Lakes).

### 2.2 Collection of blood specimens

Peripheral venous blood samples were collected as recommended by the Clinical Laboratory Standards Institute (CLSI), Document GP41.^9^ In brief, the samples were divided into two groups. The first group (first set) comprised three of the tubes taken from each participant (one plain, one plasma EDTA, and one SST). For these samples, serum and plasma were separated immediately after collection by centrifugation for 10 min at 3500 rpm. An aliquot of separated serum and plasma from each tube was immediately analyzed for IPTH according to the method described below. These provided the initial time (baseline) data. The remaining separated serum and plasma were aliquoted into plastic tubes and stored at different conditions: room temperature (24°C), fridge (4°C), and freezer (−20°C). These were subsequently analyzed at day 2, day 4, and day 8.

The second group (second set) comprised two of the tubes taken from each participant (one plain and one plasma EDTA). For these samples, separation was delayed for two h after collection. At that point, the same separation protocol was applied as for the first set, immediately followed by analysis for IPTH. The means of the IPTH content of the serum and plasma samples were compared. In addition, the means of the IPTH content of the delayed serum and plasma were compared to the initial time data, as were the means of the stored serum and plasma from the first set, in order to assess possible deterioration in stability.

To confirm that our patients were healthy, other IPTH related tests were performed using the initial blood samples. They were as follows (mean ± SD): calcium (2.46 ± 0.07 mmol/L), phosphate (1.12 ± 0.18 mmol/L), albumin (46.0 + 2.0 g/L), alkaline phosphatase (71 ± 18 U/L), and vitamin D (49.0 ± 16.0 nmol/L). The measurement of blood pressure (BP; mmHg) was taken automatically by an Accutor Plus vital signs monitor, Datascope. Data were collected using data entry sheets for both anthropometric and testing parameters. This study was approved by the institutional review board of the King Abdullah International Medical Research Center (IRB # RJ18/085/J; 25–03–2019), King Saud bin Abdulaziz University for Health Sciences, King Abdulaziz Medical City, Jeddah Saudi Arabia.

### 2.3 Laboratory Testing

The IPTH assay was done using an Architect i2000 instrument (Abbott Diagnostics). The assay is based on chemiluminescent microparticle immunoassay (CMIA) for quantitative determination of IPTH in human serum and plasma. The kit insert instructions were followed to ensure the reliability of the results. Reagents were donated by the Abbott Company (Medi‐Serve). The Architect IPTH assay was investigated for within and between run precision (percentage of coefficient of variation (%CV)). Between run CVs were 8.7%, 4.2%, and 4.1% for the low, medium, and high control samples, respectively. Within run CVs were 8.7%, 4.1%, and 4.1% for low, medium, and high control samples, respectively. The precision study was conducted based on guidance from the National Committee for Clinical Laboratory Standards Institute Protocol EP5‐A.^10^

### 2.4 Statistical analysis

Data were collected for both anthropometric and testing parameters using data entry sheets. The Statistical Package for Social Sciences (SPSS), version 25 (IBM) was utilized for data management. A test for normality was performed on all continuous variables, and was normal, as evidenced by a non‐significant Shapiro‐Wilk test. Accordingly, parametric statistical tests were adopted for comparisons. One‐way ANOVA, paired T test, ANOVA with repeated measures, Least significant difference, and percent change tests were applied, and data were reported as mean ± SD and statistical significance was determined at *p *< 0.05.

## 3 RESULTS

### 3.1 Comparison between different sample types

When plasma and serum were separated immediately after collection, the recorded IPTH mean values ± SD in the three tube types were as follows: in plasma EDTA tubes (67.3 ± 26.0 pg/mL), in SST serum tubes (65.9 ± 24.8 pg/mL), and in plain serum tubes (67.8 ± 25.9 pg/mL). One‐way ANOVA showed that there was no statistically significant for the IPTH values using these different types of tubes at the *p *< 0.05 level F (2,87) = 0.047, *p* = 0.954.

### 3.2 Effects of a two‐h delay in separation on serum and plasma IPTH levels

Delaying separation for up to two h was associated with a decrease in the IPTH value in the plasma EDTA tubes to 62.1 ± 23.6 pg/mL, when compared to that in the initial plasma samples (67.3 ± 26.0 pg/mL). A paired T test analysis indicated that this was a statistically significant decrease (*p*<0.001). The total observed error (TEo) or percentage change of the level of plasma IPTH between initial time and delayed status was −7.4%.

In addition, delaying separation for up to two h decreased the IPTH value in the serum collected into plain tubes from 67.8 ± 25.9 pg/mL to 65.1 ± 24.9 pg/mL. Again, the paired T test indicated that this was a statistically significant decrease (*p *= 0.003). The percentage change of the level of IPTH in serum between initial time and delayed status was −3.8%.

### 3.3 Effect of storing samples in different conditions, over time

#### 3.3.1 Storage of plasma

ANOVA with repeated measures was conducted in order to compare the effect of storage conditions on the measured levels of IPTH over time. It was found that, compared to the initial level, there was a statistically significant difference (*p *< 0.001) in the level of IPTH in EDTA plasma kept at room temperature (25°C) as time progressed (Day 2, Day 4, and Day 8), with the lowest level being recorded at day 8 (46.7 ± 18.8 pg/mL).

**FIGURE 1:**
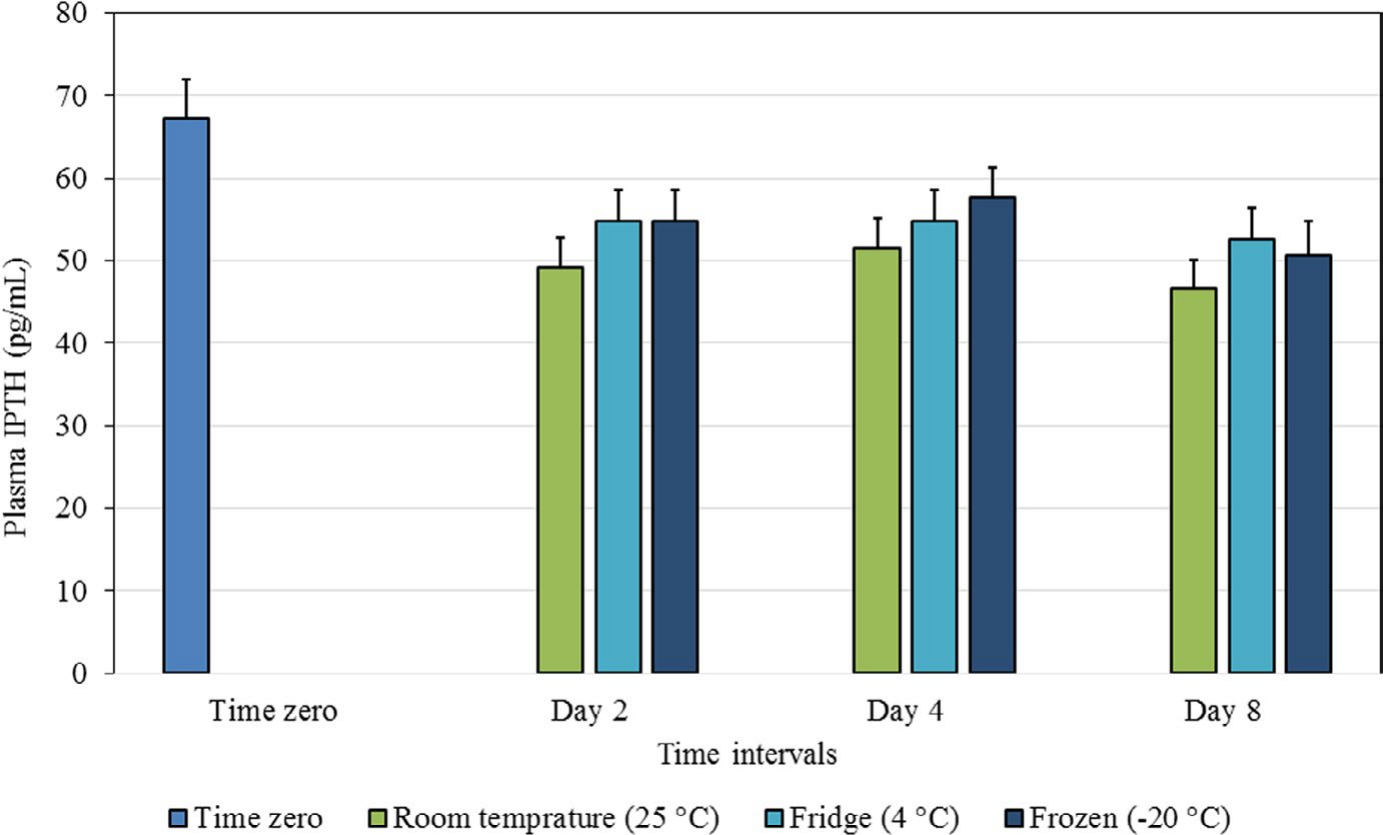
Difference between IPTH mean values ± SE in plasma stored in different conditions, over time

Similarly, there was a statistically significant difference (*p *< 0.001) in the level of IPTH in EDTA plasma kept in the fridge (4°C) as time progressed (Day 2, Day 4 and Day 8); with the lowest level again being recorded at day 8 (52.7 ± 20.9 pg/mL). The same pattern was seen when the EDTA plasma was stored in a freezer (−20°C). The decrease in plasma IPTH was again statistically significant on all days (*p *< 0.001) with the lowest level at day 8 (50.6 ± 22.9 pg/mL). Table 2 and Figure 1 show the differences in the level of IPTH plasma at each time interval for the three storage conditions.

**TABLE 2:** Comparison of IPTH mean values ± SD of IPTH in plasma and serum stored in different conditions, over time.

IPTH (pg/mL)	Initial	Day						ANOVA	
		2	%	4	%	8	%	F‐ stat	*p*‐value
Plasma (RT)	67.2 ± 25.9	49.2 ± 19.8^a^	−26.7	51.5 ± 19.6^a^	−23	46.7 ± 18.8^a^	−30.8	200.88	<0.001
Plasma (Fridge)	67.2 ± 25.9	54.9 ± 20.9^a^	−18	54.8 ± 21.4^a^	−18.4	52.7 ± 20.9^a^	−21.7	201.8	<0.001
Plasma (freezer)	67.2 ± 25.9	54.8 ± 21.0^a^	−17.9	57.7 ± 20.0^a^	−13.8	50.6 ± 22.9^a^	−23.7	208.22	<0.001
Serum (RT)	67.8 ± 25.9	37.5 ± 16.1^a^	−45	27.5 ± 13.5^a^	−29	15.3 ± 10.1^a^	−77	192.2	<0.001
Serum (Fridge)	67.8 ± 25.9	56.4 ± 21.7^a^	−17	54.2 ± 21.6^a^	−20.4	49.5 ± 19.4^a^	−27.2	201.8	<0.001
Serum (freezer)	67.8 ± 25.9	58.8 ± 22.6^a^	−13.2	60.8 ± 22.4^a^	−9.8	56.2 ± 20.9^a^	−16.6	213.7	<0.001

^a^ LSD *post hoc* test (*p* < 0.001), the mean value is significantly different when compared to the initial IPTH value. % indicates the total observed error (TEo) or change in percentage between the current condition value and the initial time value ± SD.

#### 3.3.2 Storage of serum

**FIGURE 2:**
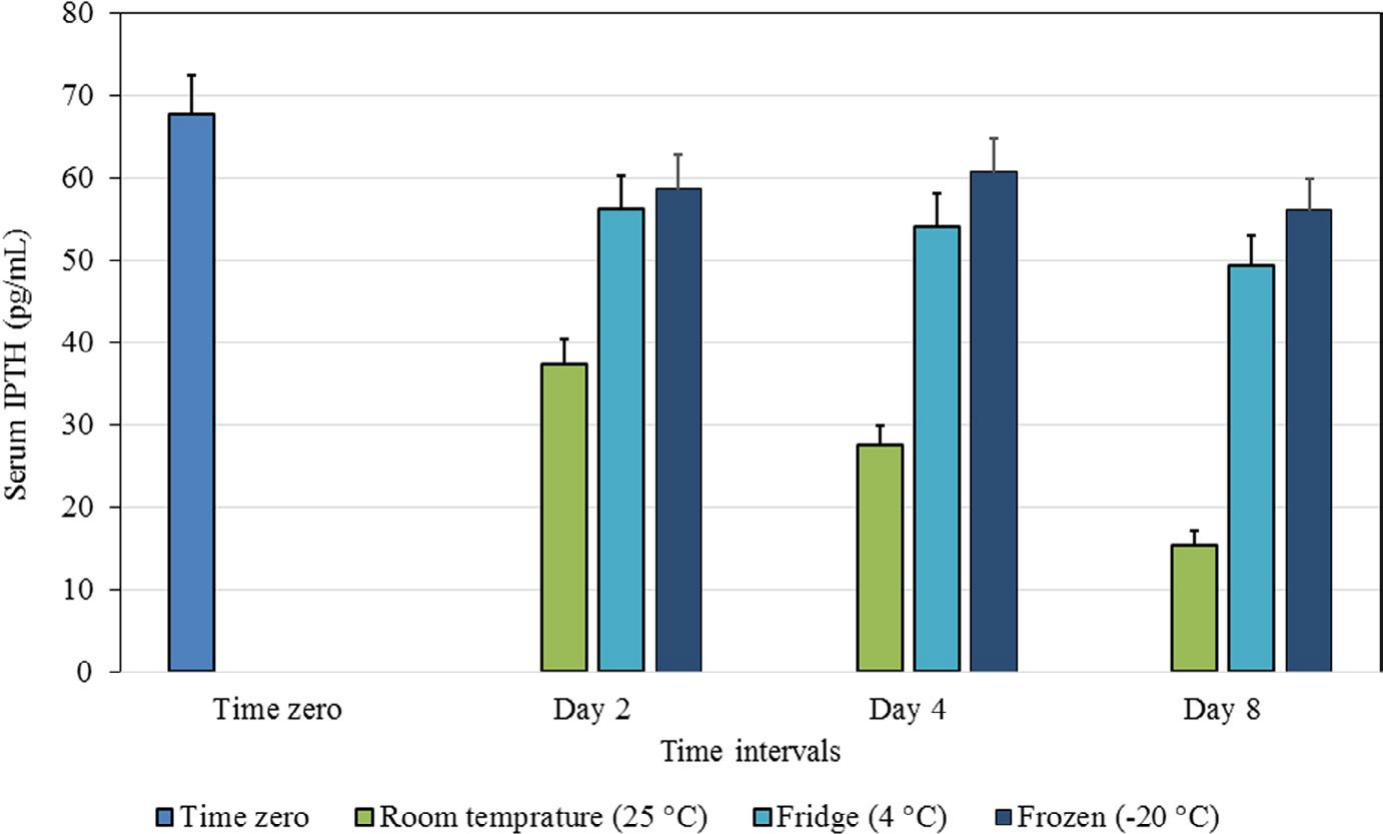
Difference between IPTH mean values ± SE in serum stored in different conditions, over time

ANOVA with repeated measures was conducted in order to compare the effect of storage conditions on the measured levels of IPTH over time. It was found that, compared to the initial time, there was a statistically significant difference (*p *< 0.001) in the level of IPTH in serum kept at RT (25°C) as time progressed (Day 2, Day 4, and Day 8) with the lowest level being recorded at day 8 (15.3 ± 10.1 pg/mL). Similarly, there was a statistically significant difference (*p *< 0.001) in the level of IPTH in serum kept in the fridge (4°C) as time progressed (day 2, day 4, and day 8) with the lowest level again being recorded at day 8 (49.47 ± 19.4 pg/mL). The same pattern was seen when the serum was stored in a freezer (−20°C). The decrease in plasma IPTH was again statistically significant on all days (*p *< 0.001) with the lowest level at day 8 (56.1 ± 20.9 pg/mL). Table 2 and Figure 2 show the differences in the level of serum IPTH at each individual time point for the three storage conditions.

### 3.4 Total allowable error (TEa) and Total observed error (TEo):

**FIGURE 3:**
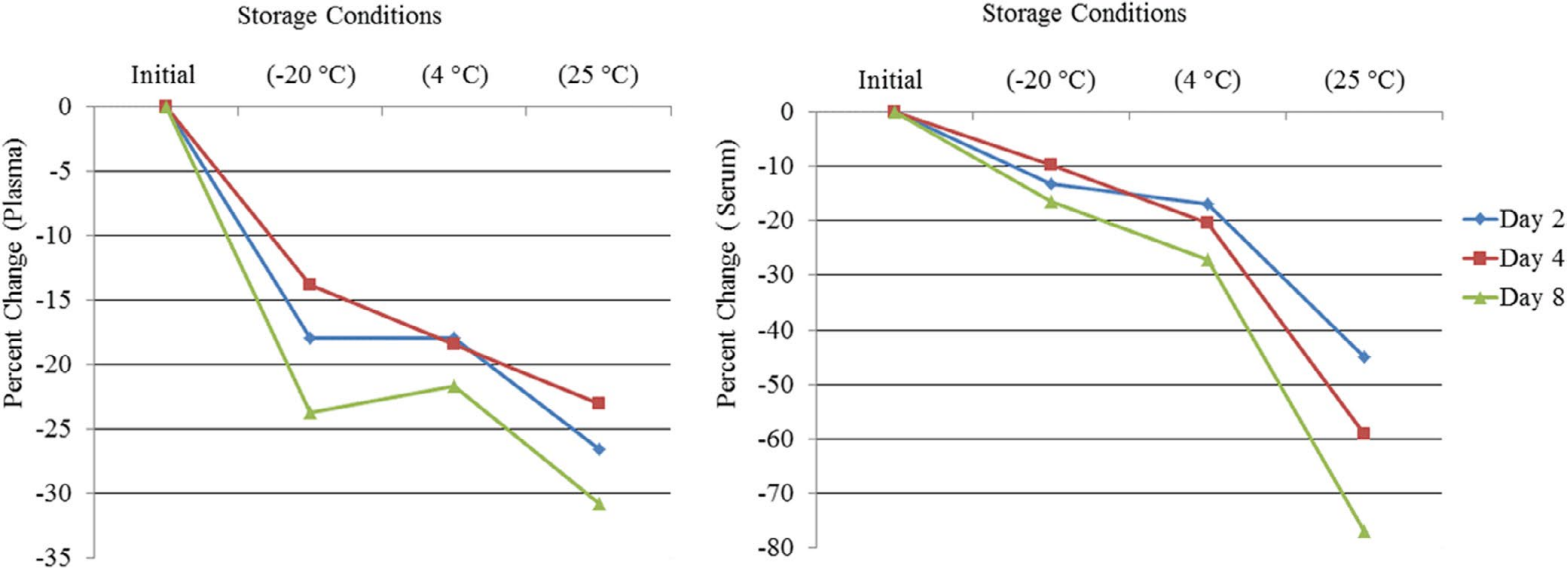
Comparison between mean percentage changes in IPTH levels in EDTA plasma and serum according to storage conditions and time interval

Data on total changes of analyte concentrations have important clinical implications in patient management. One of the most widely accepted approaches for this purpose is to compare the percentage of change (TEo) with the quality parameter called the total allowable error (TEa).^11^ TEa is a quality requirement that sets a limit for the summation of imprecision (random error) and bias (inaccuracy, or systematic error) that are tolerable in a single test result to ensure clinical usefulness. TEa can be defined as the error that is allowable when compared to the reference or original value without affecting the interpretation of the test result or exceeding the clinical decision thresholds. It can be expressed as the maximum error and imprecision which can be allowed for each analyte. Figure 3

The TEa for IPTH is 30% as recently recommended by the Clinical Laboratory Improvement Amendments.^12^ This means that observed total error (TEo) values of more than ± 30% of the reference value will be clinically significant. Thus, TEo values not exceeding the TEa have no clinical significance on patient outcomes. In this study, the IPTH value at the initial time in each condition will be taken as the reference value.

The results in Table 2 show the TEo as a change in percentage in the level of IPTH (plasma or serum) from the initial time to day 2, day 4, and day 8 at room temperature, fridge, and frozen status. For plasma, the biggest change was observed at room temperature and at day 8 (30.8%), exceeding the TEa of 30% provided by CLIA. In contrast, although there were statistically significant decreases in the IPTH values, compared to the initial time when plasma was stored in the fridge or freezer, these were not clinically significant.

With respect to stored serum, a similar picture emerged; namely, statistically significant, but clinically no significant, reductions in IPTH values on days 2, 4, and 8, compared to the initial time when serum samples were stored in a fridge or freezer. On the other hand, serum samples stored at room temperature showed a clinically significant reduction on all days; specifically: day 2 (45.0%), day 4 (59.0%), and day 8 (77.0%), clearly exceeding the TEa of 30% provided by CLIA.

## 4 DISCUSSION

Laboratory test results contribute to about 70% of all clinical decisions.^13^ Therefore, for reliable clinical decision, it is important to investigate different laboratory tests at different conditions.

Parathyroid hormone (PTH) is the main regulator for calcium and phosphorous levels in blood circulation through its direct effects on the cells of different vital organs.^14^ There are many factors that can affect the reliability of PTH results.^15^, ^16^ In this study, we provide information about IPTH pre‐analytical stability that should be useful.

We controlled subject variability by collecting samples from the same subject in all tube types at the same time. To control instrument variability, meanwhile, we analyzed the initial time samples immediately on collection using the same instrument. In practice, it is useful to have some freedom in the choice of tube type. When many tests need to be done simultaneously on a sample at the same laboratory using just a single tube helps minimize the volume of blood taken from patients, controls costs and supports the quality of results.

Our results showed that there were no statistically significant differences in IPTH levels between samples collected using plain, SST, or EDTA tubes. Interestingly, a previous study to assess IPTH levels in serum and EDTA plasma samples did indicate a statistically significant difference when an Immulite 2000 analyzer was used for analysis.^7^ That study also showed that these differences were not apparent when an Architect analyzer was used, and thus may be due to the different reagent components utilized in the two devices. In addition to the study of La'ulu and Roberts included fewer subjects than ours. Another implication of our finding is that there were no statistically significant differences between tubes containing a gel separator in the SST had no significant effect on IPTH results when compared to serum or EDTA plasma.

Overall, our findings in respect to the impact of the choice of collection tube support the use of the smallest number of tubes for blood collection that would be necessary for many clinical tests. This will allow clinical laboratories to minimize the volume of blood taken from patients, and reduce costs.

A delay in separating serum or plasma from packed cells may contribute to unreliability in IPTH results. Schleck et al. (2017) reported that EDTA blood samples for IPTH are more stable than serum when centrifugation was delayed for 18 h. In our study, we investigated the impact of a two‐h delay in the separation of serum and plasma from clotted samples collected in plain and EDTA tubes, respectively, as is common in clinical laboratories. Our results showed that there was a statistically significant reduction in IPTH values in both tube types when compared to the initial time control value (a percentage change of −7.4% for plasma and −3.8% for serum). The impact on IPTH levels, however, did not exceed the maximum TEa of 30%, and thus, although these differences were statistically significant, they were still not considered clinically significant. This finding is of great importance since many IPTH samples are collected and transported from outside clinics or dialysis centers to the main laboratories and this may cause a delay in separation. This finding may reassure physicians that the IPTH results from samples separated within two h of collection are reliable.

Most previous studies have only investigated one or two storage conditions.^4^, ^5^, ^6^, ^7^, ^16^, ^17^ Indeed, no previous studies have included all three storage conditions and assessed the effect of time intervals on IPTH at the same time. Overall, our results showed that the IPTH results for samples stored at different conditions (room temperature, 25°C, refrigerator, 4°C, and freezer, −20°C) were significantly different when compared to the initial time for both plasma and serum across all of our selected storage times.

Although the differences we found in IPTH levels arising from storage conditions were statistically significant they were not all clinically significant. Specifically, our study showed that, at RT, while the mean observed total error (TEo) for serum samples exceeded the TEa of 30% for all subsequent days, plasma samples exceeded the TEa only on day 8. This means that plasma samples are more stable in room temperature storage and this is compatible with the outcomes of previous studies.^4^, ^5^, ^6^, ^7^, ^16^

In addition, we found that, when samples were stored in a refrigerator, the observed total errors (TEo's) did not exceed the TEa of 30%. This means that, regardless of the sample type, IPTH can be stored in the fridge for up to 8 days without any clinically significant changes.,^7^ in contrast, suggested that IPTH samples could be stored in a fridge for only 24 h only before the deterioration in IPTH levels became clinically significant. The different conclusions we arrive at here, however, mainly reflect the fact that we used the TEa value of 30% as the threshold for clinical significance (as recommended by CLIA), whereas La'ulu and Roberts (2010) used the desirable analytical error limit, which is only 12.5%.

In addition, our study showed that when samples were stored in a freezer, none of the TEo values exceeded the recommended TEa. While one previous study suggests that IPTH can be stored in a freezer for up to 5 days before deterioration becomes clinically significant,^6^ our results extend that period to 8 days, for both serum and plasma IPTH samples.

Although in this study we did not investigate the desirable quality specifications components but they can be estimated based on within and between individuals variations as described by Fraser and Harris.^11^ The desirable specifications were summarized from previous studies by Ricos.^18^ The IPTH components were not included in the listed summary of Ricos but they were recently published.^19^

Our study does have some limitations, however. Firstly, it is limited to a specific type of instrument, the Architect i2000. Although most analyzers have the same principle of analysis, they use different reagent antibodies and accordingly operate on different specifications for IPTH detection. In addition, although the Becton Dickenson collection tubes utilized in our study are widely used, tubes from different companies lead to different outcomes. The final limitation of our study was that it was conducted on healthy individuals only rather than on patients with pathological conditions or dialysis.

In conclusion, we show that IPTH is more stable than previous research suggests. IPTH can be tested using plasma or serum samples with and without gel. A delay between sample separation and analysis is acceptable, but the most accurate results are obtained if IPTH is analyzed immediately after blood collection. If this is not possible, storage in a fridge or freezer would be recommended (for up to 8 days).

In general, plasma IPTH has more stability than serum and can be stored at room temperature for up to 8 days.

## ACKNOLEDGEMENTS

We are grateful for the kind financial support and collaboration received from the Saudi Society for Clinical Chemistry (SSCC). We are also grateful for the kind support from the Deanship of Research in King AbdulAziz University, King Abdullah International Medical Research Center (KAIMRC), and King Saud bin Abdulaziz University for Health Sciences (KSAU‐HS), Jeddah, Kingdom of Saudi Arabia.

## CONFLICT OF INTERESTS

The authors declare no competing interests.

## AUTHOR CONTRIBUTIONS

HK researched the literature and conceived the study. Recruitment of subjects and analytical work was carried out by HK, HF, and MD. Data analysis was done by FB. AB, SB, GF, and AM reviewed all drafts of the manuscript. All authors reviewed and edited the manuscript and approved the final version of the manuscript.

## DECLARATIONS

The authors have no declarations.

## ETHICAL APPROVAL

Ethical approval for this study was approved by the institutional review board of King Abdullah International Medical Research Center (IRB # RJ18/085/J; 25‐03‐2019), King Saudi bin Abdulaziz University for Health Sciences (KSAU‐HS), Jeddah, Saudi Arabia.

## Data Availability

The data used to support the findings of this study are available from the corresponding author upon request.
